# Improved Processing and Properties for Polyphenylene Oxide Modified by Diallyl Orthophthalate Prepolymer

**DOI:** 10.3390/polym11122016

**Published:** 2019-12-05

**Authors:** Honghua Wang, Qilin Mei, Yujie Ding, Zhixiong Huang, Minxian Shi

**Affiliations:** School of Materials Science and Engineering, Wuhan University of Technology, 122 Luoshi Road, Wuhan 430070, China; 13343434177@163.com (H.W.); dingyujie26@126.com (Y.D.); zhixionghuang@whut.edu.cn (Z.H.)

**Keywords:** diallyl orthophthalate prepolymer, polyphenylene oxide, processibility, morphology, phase separation

## Abstract

Diallyl orthophthalate (DAOP) prepolymer was investigated as a reactive plasticizer to improve the processability of thermoplastics. The rheology of blends of DAOP prepolymer initiated by 2,3-dimethyl-2,3-diphenylbutane (DMDPB) and polyphenylene oxide (PPO) was monitored during the curing process, and their thermal properties and morphology in separated phases were also studied. Differential scanning calorimetry (DSC) results showed that the cure degree of the reactively plasticized DAOP prepolymer was reduced with increasing PPO due to the dilution effect. The increasing amount of the DAOP prepolymer led to a gradual decrease in the viscosity of the blends and the rheology behavior was consistent with the chemical gelation of DAOP prepolymer in blends. This indicated that the addition of the DAOP prepolymer effectively improved processability. The phase separation occurring during curing of the blend and the transition from the static to dynamic mode significantly influences the development of the morphology of the blend corresponding to limited evolution of the conversion around the gel point.

## 1. Introduction

Thermoplastics such as polyphenylene oxide (PPO), polyvinyl chloride (PVC) and polycarbonate (PC), possess a range of desirable properties including high strength, stiffness, toughness and excellent dimensional stability [1]. However, their processabilities of extrusion, injection and rotation are often constrained due to their high melt viscosity [2]. Raising the temperature will lead to degradation of engineering plastics such as rigid PVC, polyphenylene oxide whose thermal decomposition temperature is lower than the molding temperature. Moreover, the laminated nanoparticles can greatly improve the mechanical properties of the thermoplastics; however, the addition of such additives will further increase the melt viscosity [3,4,5].

In recent years, various methods have been tried to reduce the processing temperature, including lowering molecular weight of thermoplastics, broadening the molecular weight distribution, adding a low molecular weight plasticizer or blending thermoplastics with more processable plastics. Nevertheless, all of these initiatives are at the penalty of mechanical properties and the decrease of heat distortion temperature [6,7].

Recently, a few researchers have reported the application of cross-linkable monomers as plasticizers to improve the processability of thermoplastics. Unlike other plasticizers, the reactive thermosetting monomers have a lower viscosity and they can be polymerized into a cross-linked phase at the end of the processing [8,9,10]. Therefore, the reactive monomers can effectively lower the processing temperature and finally retain thermodynamic properties of plastics. Accordingly, most of the previous research on the application of reactive monomers for thermoplastics used diallyl orthophthalate (DAOP), because DAOP has a high boiling temperature (290 °C) [11], a relatively low reaction rate due to its degradative chain transition reaction and cyclization [12,13,14,15], a high gel point (170 °C) up to which DAOP can be processed without any difficulties, and a relatively good compatibility with thermoplastics.

Although some progress improving processability of thermoplastics by DAOP cross-linkable monomers has been made in the state-of-the-arts [2,7,13], many problems still exist in the specific implementation process. For instance, few cross-linkable reactive plasticizers are suitable for processing conditions of thermoplastics; most of them are in their liquid state which is easy to volatilize in the molding process. Moreover, in order to ensure the molding process we usually used inactive monomers s, such as dioctyl phthalate (DOP) and aliphatic ester, which resulted in monomers in blends. Thus, many small molecule droplets of monomers were always left. This corresponds to adding plasticizer with a low molecular weight which decreases the mechanical properties and heat distortion temperature of thermoplastics. In view of these weaknesses, this paper used DAOP cross-linkable prepolymer to improve the processability of thermoplastics. Compared to DAOP cross-linkable monomers, the DAOP prepolymer with a relatively low degree of polymerization also has the existing advantages that the DAOP monomer has. A preferable DAOP prepolymer is in its solid state at room temperature and does not generate gas during curing which may not cause a mass loss of DAOP in the blend. The DAOP prepolymer can also be molded at low pressures which may reduce equipment requirements. In addition, the molecular weight of the DAOP prepolymer polymerized with 2,3-dimethyl-2,3-diphenylbutane (DMDPB) is about 4000 to 7000 g/mol which is higher than monomers. Hence the cross-linking degree will also increase after the same processing. Moreover, with a high initiation temperature of DMDPB, DAOP prepolymer would be kept in storage for a certain period of time at room temperature even with residual initiator in blends. Since the prepolymer has a certain cross-linking degree, residual unreacted DAOP prepolymer has a insignificant impact on the thermodynamic properties of the blend. Although the melt viscosity of the DAOP prepolymer is higher than that of the DAOP monomer, it is still far below the melt viscosity of thermoplastics. Furthermore, the molecular weight of the DAOP prepolymer polymerized with DMDPB is much lower than the DAOP prepolymer of the market supply with an average molecular weight of about 60,000 g/mol. The DAOP prepolymer is therefore expected to effectively lower the processing temperature and finally to retain the thermodynamic properties of plastics. Thereby, the DAOP prepolymer can overcome the shortcomings of the DAOP monomer in improving the processability of thermoplastics.

In this paper we used DAOP prepolymer as a reactive plasticizer initiated by DMDPB for improving PPO processing. The molecular weights and their distributions of the DAOP prepolymer initiated by DMDPB at various temperatures were investigated. The curing mechanism of DAOP initiated by DMDPB was studied by infrared spectroscopy (IR). The curing behavior of the DAOP prepolymer in the presence of various PPO contents are investigated by means of dynamic differential scanning calorimetry (DSC), and the rheological properties of PPO with increasing the DAOP prepolymer content in the PPO/DAOP prepolymer blend are also observed by the changes in the viscosity and modulus. In addition, the morphological developments of the blends based on the thermoplastic matrix with a reactive system undergoing polymerization were studied with the system consisting of 60 wt% of PPO and 40 wt% of the DAOP prepolymer by scanning electron microscopy (SEM). The transition from the static to the dynamic mode under shearing reveals the influence of the shearing effect on the development of the morphology around the gel point. These investigations of the properties provide the required information for the application of such reactive DAOP prepolymers to improve the processing of PPO.

## 2. Experimental

### 2.1. Materials

The diallyl orthophthalate (DAOP) prepolymer was made in the laboratory. The initial monomer DAOP was here purchased from Sinopharm Chemical Reagent Co. Ltd., Shanghai, China. 2,3-dimethyl-2,3-diphenylbutane (DMDPB) was provided by Sinopharm Chemical Reagent Co. Ltd., Shanghai, China and used as the initiator to further polymerize the DAOP prepolymer. The polyphenylene oxide (PPO) powder was supplied by Bluestar Rui Cheng foster chemical Co. Ltd., Beijing, China and grades for LXR-40.

### 2.2. Sample Preparation

The DAOP monomer was added into a glass three-necked flask under a nitrogen atmosphere (nitrogen was passed below reaction surface) up to a specified temperature of 200 °C and then initiator was added with a mass ratio of 3%. The mixture was maintained with stirring for a certain time and a homogeneous solution called DAOP prepolymer was obtained which consisted of monomer, polymer and DMDPB. The samples to measure the molecular weights DAOP prepolymer were taken at a variant time in the curing process. The samples for infrared spectroscopy (IR) analysis of DAOP prepolymer were obtained at a variant curing time.

The PPO/DAOP prepolymer blends were prepared by an internal mixer. The proportion of the blends are identified by quality, e.g., the 60 PPO/40 DAOP prepolymer identifies the blend composed of 60 wt% PPO and 40 wt% DAOP prepolymer. For differential scanning calorimetry (DSC) of the thermal behavior and rheological properties of the PPO/DAOP prepolymer blends, the samples were prepared by manually mixing DAOP prepolymer with PPO to form a homogeneous phase. The samples of mechanical properties of PPO/DAOP prepolymer blends were prepared by melt-blending at 200 °C for 5 min and then hot-pressing process at 200 °C for 2 h.

The morphology of the PPO/DAOP prepolymer blends processing under static and dynamic conditions were studied by SEM. The samples of static condition were prepared by manual mixture and then in a drying oven at 200 °C for 30 min, while the samples of dynamic condition were prepared by an internal mixer (SU-70, made by Suyan Tech., Changzhou, China) at 200 °C for 30 min. Furthermore, the samples, for investigating the system phase separation influenced by synergy of shear and gelation reaction, were obtained by using a mixer rotor to apply shear force at a certain time (before, during and after the gelation time) in an internal mixer.

### 2.3. Instruments

The molecular weights of DAOP prepolymer were measured by Gel permeation chromatography (GPC) by an Agilent 1100 system, made in Santa Clara, CA, USA, with tetrahydrofuran of 0.4 mL/min as mobile phase. IR spectra were recorded on KBr pellets from 4000 to 400 cm^−1^ by a Nicolet Nexus IR Spectra, made in Madison, WI, USA. The DSC studies were conducted on the blends with various contents of PPO in hermetically sealed pans. The heating and cooling experiments were performed at 10 °C /min with Perkin Elmer DSC7, made in Waltham, MA, USA. The sample (10 mg) was sealed under nitrogen in aluminum pans. Temperature ramping DSC studies during curing were performed from 50 to 350 °C. The rheological properties of the PPO/DAOP prepolymer blends were measured using a Malvern 200, made in Marvin, UK, in the parallel plate mode at 220 °C. The average strains varied with the storage modulus (G’), ranging from 100% for G’ < 102 Pa to 1% for G’ > 105 Pa. The mechanical properties were measured by RGM-4100 electronic material testing system, made in Shenzhen, China. The morphology of the blends was studied by examining the fracture surfaces using JSM-IT300 Scanning Electron Microscope (SEM), made in Tokyo, Japan. The specimens were freeze-fractured using liquid nitrogen and then sputter-coated with silver and mounted on a carbon tape prior to the SEM examination.

## 3. Results and Discussion

### 3.1. Synthesis of the DAOP Prepolymer

**Figure 1:**
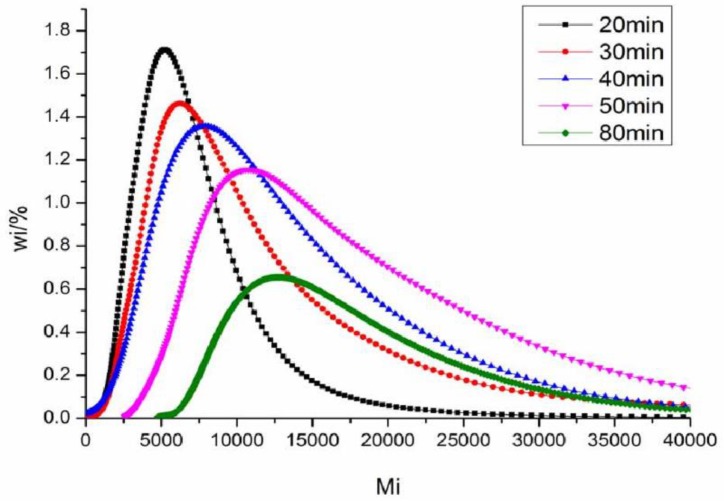
Molecular weight distribution of DAOP prepolymer at different reaction temperatures.

The results of molecular weight and its distribution of DAOP prepolymer at different reaction temperatures are shown in Table 1 and Figure 1. The results show that the average molecular weight of the DAOP prepolymer is about 4000 to 7000 g/mol. As polymerization time increased, the molecular weight of the prepolymer gradually increased. The molecular weight is much lower than that of the market-sold DAOP prepolymer with an average molecular weight of about 60,000 g/mol. According to the Mark-Houwink-Sakurada equation, at a constant temperature, the intrinsic viscosity of the polymer solvent system and the molecular weight of the polymer accord with the Equation (1)

[η] = KM^α^, (1)

where [η] is intrinsic viscosity, M is molecular weight, K and α are constants.

It can be concluded that the intrinsic viscosity of the system increases with increasing molecular weight. With a lower molecular weight compared to market-sold versions, the DAOP prepolymer is more helpful in improving the processability of thermoplastics as a reactive plasticizer. 

**Figure 2:**
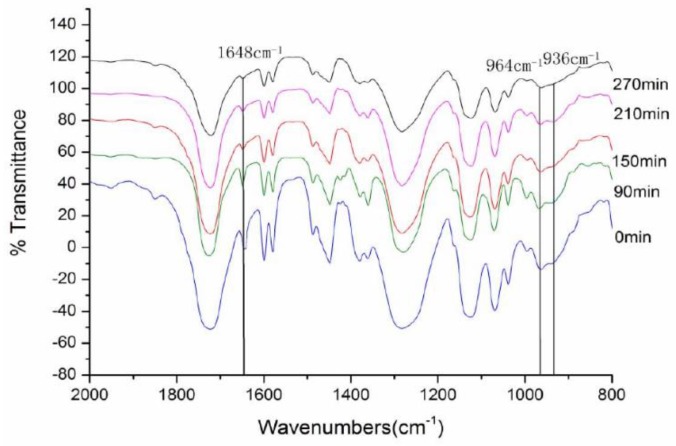
IR spectra of DAOP prepolymer at different curing times.

**Figure 3:**
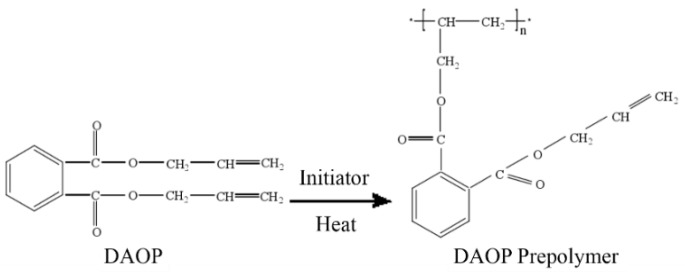
The curing procedure of DAOP.

The IR spectra of DAOP prepolymer at different curing times from 0 min to 270 min was recorded to investigate the reaction mechanism in the curing process. As shown in Figure 2, the absorptions at 1648 cm^−1^ and 934 cm^−1^ assigned to C=C gradually decreased with increasing time. On the basis of IR spectra, it is concluded that the polymerization mechanism is the addition reaction of C=C as shown in Figure 3.

### 3.2. Cure of the DAOP Prepolymer/PPO Blends

**Figure 4:**
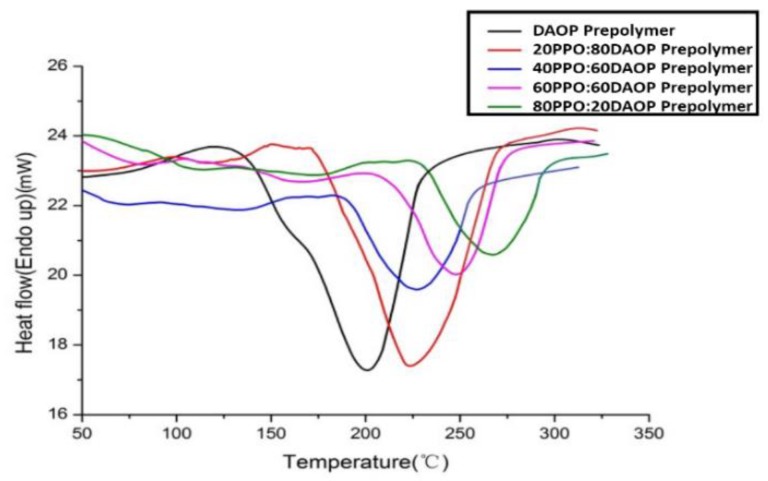
Dynamic differential scanning calorimetry (DSC) curves of PPO/DAOP prepolymer blends.

DSC curves were obtained to investigate the PPO/DAOP prepolymer blends with various ratios in order to determine the effect of PPO on the curing of DAOP prepolymer. It can be seen in Figure 4 and Table 2 that the peak temperatures (*T*_p_) of the blends were shifted from 203 °C to a higher temperature of 264 °C with increasing PPO; this may be triggered by the dilution effect. Because the presence of PPO will reduce the concentration of reacting allylic functional groups, the rate of polymerization will thus be expected to slow down. Table 2 also shows that the increase in PPO reduces the heat of polymerization. This may be explained as that the DAOP prepolymer concentration in the matrix lowered with increasing PPO and became more difficult to link the prepolymer units into the poly-DAOP structure. When more PPO were added to the blended, the concentration of the initiator in the matrix is also reduced, which may also reduce the conversion due to a premature loss of the initiator via dead-end polymerization or the degradation process.

### 3.3. Rheological Properties during Cure of DMDPB/DAOP Prepolymer/PPO Blends

**Figure 5:**
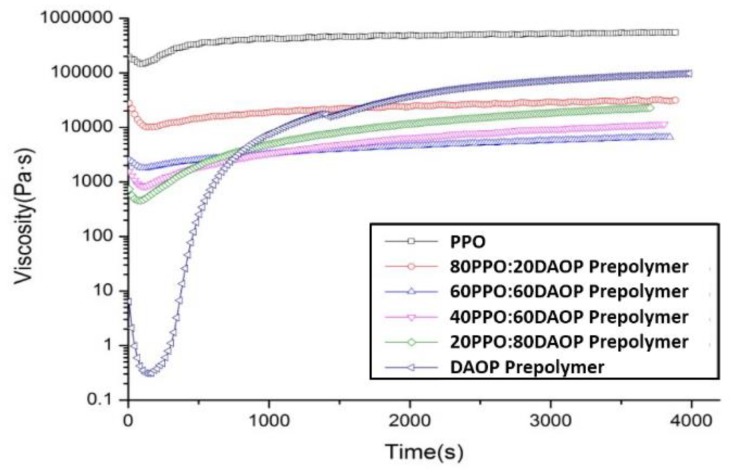
Viscosity of 2,3-dimethyl-2,3-diphenylbutane (DMDPB)/DAOP prepolymer/polyphenylene oxide (PPO) blends during cure.

The evolution of viscosity, as shown in Figure 5, of the DAOP prepolymer and PPO/DAOP prepolymer blends were measured at 220 °C. At the commencement of the reaction it can been seen that the viscosity of the neat DAOP prepolymer is very low which indicates that the degree of polymerization of DAOP prepolymer is relatively low and is able to be further polymerized. Then the viscosity increased suddenly at about 400 s and tended to be of a considerably high value where the DAOP prepolymer began to gel. Similarly, with different ratios of PPO, the blends also have a certain degree of gel reaction; however, with the increase of PPO the gelation did not occur obviously like the neat DAOP prepolymer, caused possibly by phase separation of PPO into a dispersed phase which reduced the concentration of the polymer in the continuous phase and thus reduced the increase rate of viscosity. Therefore, the viscosity of blends with higher ratios of PPO such as PPO/DAOP prepolymer (80/20, 60/40) increased more gradually with time and then reached a plateau. However, in general, with increased DAOP prepolymer, the initial viscosity of the blends was reduced obviously. The viscosity of DAOP prepolymer increases more rapidly than the other and reaches a plateau which is higher than the PPO/DAOP prepolymer and lower than pure PPO. DAOP prepolymer is capable of taking a cross-linking reaction to form a three-dimensional network structure and finally leading to a higher viscosity than others, of which cross-linking degrees are decreased by dilution effects. Furthermore, the intrinsic viscosity of PPO is very huge. The addition of DAOP prepolymer to PPO can also retain the thermodynamic properties of PPO to some extent by polymerizing the DAOP reactive plasticizer after the processing operation. In addition, the rheological results indicated that the polymerization of the DAOP prepolymer occurring in the blends in the processing operation retained the thermodynamic properties of PPO to some extent.

**Figure 6:**
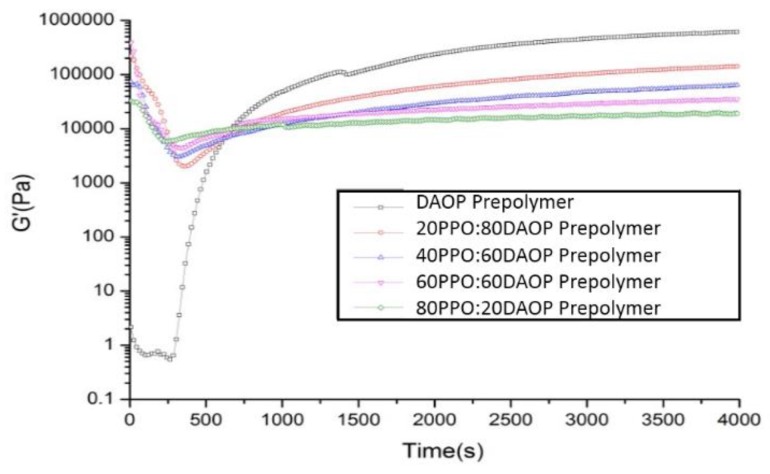
Variation of modulus with time of PPO/DAOP prepolymer blends.

Figure 6 presents the evolution of the storage modulus (G’) of the DAOP prepolymer and PPO/DAOP prepolymer blends during curing. The data for DAOP prepolymer shows an abrupt rise in about 400 s, which indicates the blend has undergone an obvious gelation at this point, thus a remarkable rise in G’ during the gelation region has occurred. It should be noted here that 1 MPa was typical for a lightly cross-linked rubber and reached a rubbery plateau from the beginning of the viscous state. With the increase of PPO, however, the trends of G’ exhibited a more gradual increase, especially for the blends with higher levels of PPO. Although the G’ still increased from low to high values with the increase of time which confirmed that polymerization was occurring (in agreement in DSC experiments), no abrupt rise in G’ was found as compared to the DAOP prepolymer. This may be explained by the fact that the PPO has a high initial viscosity of about 500,000 Pa·s (as shown in Figure 3) and the presence of PPO leads to a substantial rise of the initial modulus of the blends resulting in a more gradual upward trend. On the other hand, DAOP prepolymer is capable of taking a cross-linking reaction to form a three-dimensional network structure and finally leading to a higher G’ than others, which is consistent with the above results. Hence, the dynamic properties like G’ can primarily be determined by using the homogeneous matrix phase which also causes a slow rise in the modulus of the polymer. 

The mechanical properties of PPO/DAOP prepolymer blends, as shown in Table 3, were measured to identify the influence of different DAOP prepolymer contents on blends. It can be concluded from Table 3 that the tensile and flexural strengths of blends decrease with more DAOP prepolymer when the contents of DAOP prepolymer are less than 20 wt%. With more DAOP prepolymer added to blends, the tensile and flexural strengths increase and maximize with a value of 62.7 MPa and 78.0 MPa in the content of 40 wt%. The reason for this result may be that less content of DAOP prepolymer results in a lower molecular weight DAOP polymer because of lower concentration of DAOP prepolymer in the system. With content increasing to 40 wt%, DAOP prepolymers tend to generate a higher molecular weight DAOP polymer to strengthen the system.

### 3.4. Synergy of Shear and Gelation Reaction on Morphology Development of Blends

**Figure 7:**
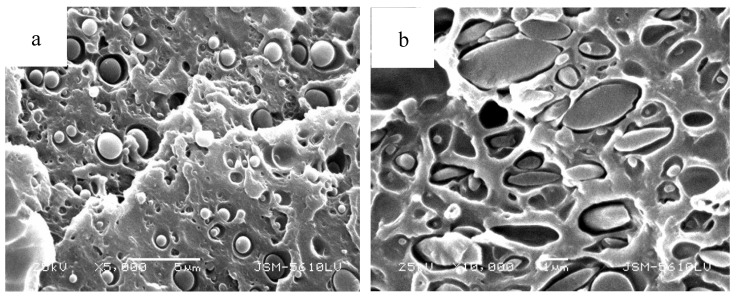
Final morphology of the 60PPO/40DAOP prepolymer blend after the polymerization of the DAOP prepolymer at 200 °C. (**a**) In static conditions, in a drying oven. (**b**) In dynamic conditions, in the internal mixer.

Since the synergy of shear and gelation reaction has a great impact on the phase morphology of the blending product, they will significantly affect the properties of the product [16,17]. The shear force is provided by rotor of internal mixer. Dynamic condition means there is a shear force in the process while static condition means no shear force. Figure 7 gives the morphological structure of the blend PPO/DAOP prepolymer (60/40) observed by using SEM under the static and dynamic conditions (for 30 min) at 200 °C. In static conditions, the blend containing 60 wt% of PPO consisted of a dispersion of spherical thermoset particles in a thermoplastic matrix. With regard to the dynamic conditions with shearing during the entire process (Figure 7b), the blend was composed of large irregular particles of the cross-linked DAOP. The shape of the DAOP polymer remains spherical eventually without shearing [18,19].

**Figure 8:**
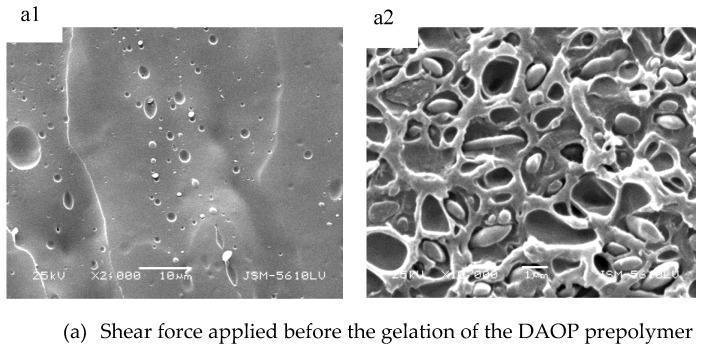

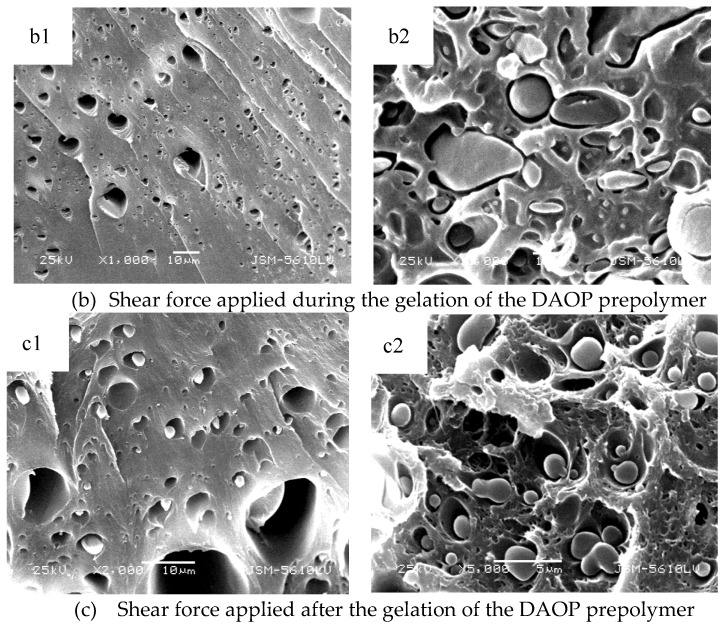
Scanning electron microscopy (SEM) of the 40 PPO/60 DAOP prepolymer blends. The indice 1 on the left indicates the morphologies obtained at the end of the static step. The indice 2 on the right indicates the final morphology after reaction in static, then in dynamic.

To clarify the effects of synergy of shear and gelation reaction on the morphological development of thermosetting/thermoplastic blends, Figure 8 shows the morphology evolution of the dispersed phase at three different situations. Similar to the results obtained from Section 3.3, the gelation time of DAOP prepolymer is about 400 s. Shear force is applied at three time points, which respectively are before, during and after the gelation time. For the three situations, the morphologies were obtained in static conditions (a1,b1,c1) and then shear force was applied by using a mixer rotor to get final morphologies (a2,b2,c2). Comparing a1, b1 and c1, the morphologies of DAOP prepolymer obtained before shearing are all at spherical dispersion phase as expected because the polymerization of the DAOP prepolymer and the phase separation proceeded in static. However, it can be found that the final morphology exhibited a difference. Figure 8(b2) shows the same as that obtained in the purely dynamic situation (Figure 7b). This implies that the polymerization of DAOP proceeded in the absence of shear before gelation led to indifference of the shape and size of the final DAOP prepolymer particles. However, the morphology observed with introducing the shear at the gel point is more complex. The morphology of the blend consisted of two kinds of particles: spherical and irregular shapes. The large and irregular particles are produced by agglomeration, and the remaining spherical particles may be owing to ineffective collisions with other particles resulting in the particles with inadequate time to agglomerate [20]. In contrast to the shear introduced before gelation, the morphology obtained when the mixer rotor started after the gel point (Figure 8(c2)) is the same as the morphology obtained in the purely static situation (Figure 7a). It means that the shear force introduced after gelation has very little influence on the morphology. It can be explained by the fact that after the gel the particle acquires a viscoelastic character which results in its interface tending to an immobile interface and thus it is difficult for the particles to agglomerate and the morphology of the dispersed phase tends to be some extent stabilized. Hence, to improve the quality of the morphology of the DAOP prepolymer/PPO blend, the shear should be avoided during the gelation process in order to reduce the formation of the irregular and large particles.

The mechanical properties of final 60PPO/40DAOP prepolymer blends which are processed by applying shear force at different time points, as shown in Table 4, were measured to identify the influence of synergy of shear and gelation reaction on blends. It can be concluded from Table 4 that the tensile and flexural strengths of blends when shear force is applied after gelation are higher than others. These results are induced by spherical particles with a better dispersion reinforcing the blends which are in accordance with the aforementioned results.

## 4. Conclusions

The DAOP prepolymer polymerized with DMDPB whose molecular weight is about 4000 to 7000 g/mol at its solid state at room temperature will have a higher cross-linking degree after the same processing. It will thus be a better approach to improve the processability of thermoplastics as a reactive plasticizer than the DAOP monomer. Even if the residual unreacted DAOP prepolymer remains, the prepolymer has a certain degree of cross-linking which will make little impact on the thermodynamic properties.

The curing behavior of the DAOP/PPO blends with DMDPB was also studied by dynamic DSC. The polymerization rate was reduced with increasing ratios of PPO, probably due to the dilution effect. The heat of polymerization was also reduced with increasing PPO.

Rheology studies of the DAOP prepolymer/PPO blends show that the DAOP prepolymer can effectively reduce viscosity and improve the processing of thermoplastics. The viscosity of the blends was reduced obviously with increasing the DAOP prepolymer and the gel of the blends with various ratios of PPO also occurred in the reaction progresses, which indicates that the polymerization of the DAOP prepolymer can occur in the blends during the processing operation and can retain the thermodynamic and mechanical properties of PPO to some extent.

The SEM observations of the DAOP prepolymer/PPO blends in various situations were carried out to evaluate the effects of gelation and shear on the morphology development of thermosetting/thermoplastic blends. The observation of the morphology before and after the transition from the static to the dynamic mode showed that the shear drastically influenced the development of the morphology around the gel point and mechanical properties. In order to improve the quality of the morphology of a phase-separated structure with dispersed DAOP particles, the shear should be avoided during the gelation process to reduce the formation of irregular and large particles.

## Figures and Tables

**Table 1:** Molecular weight of diallyl orthophthalate (DAOP) prepolymer at different reaction times.

Initiator Content	20 min	30 min	40 min	50 min	80 min
*M*_n_	4116	5049	5231	5749	6787
*M*_w_	5969	7836	8609	11,056	17,634
*M*_n_/*M*_w_	1.38	1.55	1.65	1.92	2.60

**Table 2:** DSC results of blends for various concentrations of DAOP prepolymer.

Blend	0/100	20/80	40/60	60/40	80/20
Δ*H* (J/g)	139	137	75	53	57
*T*_peak_ (°C)	203	221	226	249	264

**Table 3:** Mechanical property results of blends for various concentrations of DAOP prepolymer.

Mechanical Property	0/100	20/80	40/60	60/40	80/20	100/0
Tensile Strength/MPa	52.3	53.5	58.3	62.7	55.2	65.0
Flexural Strength/MPa	67.5	70.2	74.2	78.0	73.95	82.6

**Table 4:** Mechanical property results of final blends for different time points of applying shear force.

Mechanical Property	Before	During	After
Tensile Strength/MPa	51.3	53.5	56.3
Flexural Strength/MPa	68.8	70.2	72.1
